# miR-429 suppresses tumor migration and invasion by targeting CRKL in hepatocellular carcinoma *via* inhibiting Raf/MEK/ERK pathway and epithelial-mesenchymal transition

**DOI:** 10.1038/s41598-018-20258-8

**Published:** 2018-02-05

**Authors:** Chunmei Guo, Dongting Zhao, Qiuling Zhang, Shuqing Liu, Ming-Zhong Sun

**Affiliations:** 1 Department of Biotechnology, Dalian Medical University, Dalian, Liaoning 116044 China; 2 Department of Biochemistry, Dalian Medical University, Dalian, Liaoning 116044 China

## Abstract

Tumor metastasis is one of the main causes of hepatocellular carcinoma (HCC) high mortality. CRKL (v-crk sarcoma virus CT10 oncogene homologue (avian)-like) play important roles in tumor metastasis, however, the exact role and underlying mechanism of CRKL in HCC is still unknown. In our study, we demonstrated miR-429 negatively regulated CRKL expression *via* selectively binding to *CRKL*-3′-UTR at 3728–3735 bp site by post-transcriptionally mediating its functionality. Re-expression and silencing of miR-429 was remarkably effective in suppressing and promoting HepG2 cell migration and invasion *in vitro*. Knockdown or overexpression of CRKL exhibited similar effects as the overexpression or silencing of miR-429, whereas, CRKL overexpression (without the 3′-UTR) abrogated miR-429-induced inhibition on HepG2 migration and invasion. Moreover, miR-429-CRKL axis affected HepG2 migration and invasion potentials by regulating the adhesion ability, cytoskeleton F-actin expression and arrangement of HepG2. Furthermore, interference of Raf/MEK/ERK pathway and EMT contributed to miR-429-CRKL axis mediated metastasis inhibition. Nevertheless, miR-429 could not inhibit HepG2 proliferation through CRKL/c-Jun pathway. Taken together, our data demonstrated that miR-429 might function as an antimetastatic miRNA to regulate HCC metastasis by directly targeting CRKL *via* modulating Raf/MEK/ERK-EMT pathway. The newly identified miR-429-CRKL axis represents a novel potential therapeutic target for HCC treatment.

## Introduction

Hepatocellular carcinoma (HCC) is one of the most common cancers worldwide^1,2^. Tumor metastasis is the major problems leading to its high recurrence with low post-surgical 5-years’ survival and high mortality^3–5^. It is a multistep process, including the invasion of extracellular matrix (ECM), intravasation, translocation, migration and invasion of a secondary site, and finally the formation of metastatic nodules^3–5^. Deep study on the molecular mechanisms of HCC metastasis can get the novel therapeutic targets and improve the prognosis for HCC patients.

microRNAs (miRNAs) are 18–24 nucleotides small non-coding RNAs that regulate gene expression by directly degrading mRNA or suppressing post-transcriptional protein translation by binding to the 3′-untranslated region (3′-UTR) of targeted mRNAs^6,7^. miRNAs are involved in crucial biological processes, including cell development, differentiation, apoptosis, proliferation, metastasis and metabolism^8^. miRNAs dysregulation is involved in a wide range of human cancers, functioning as tumor promoter or suppressor in tumorigenesis, tumor progression and metastasis^9–11^.

miR-429, a member of miR-200 family, is located on chromosome 1^12^. Accumulated evidences have indicated that miR-429 dysregulation is involved in the epithelial-mesenchymal transition (EMT), progression, development, invasion, metastasis, apoptosis and drug resistance of a variety of cancers^13–16^. miR-429 is relevant to tumorigenesis in a tumor type-specific pattern, which might specifically function either as a tumor suppressor or tumor promoter candidate for certain cancers depending on the particular type of tumor cells/tissues^17–19^.

CRKL (v-crk sarcoma virus CT10 oncogene homologue (avian)-like), a member of CRK adapter protein family, is ubiquitously expressed and conserved across eukaryotic organisms^20^. It is composed by one NH2-terminal Src homology2 (SH2) domain, one N-terminal SH3 (SH3N) and one C-terminal SH3 (SH3C) domains^21,22^. CRKL has a variety of linkages for a couple of docking and proline-rich proteins BCAR1, GAB, ABL-1, Pax, GEF, C3G, BCR-ABL and SOS to form timely and localized complexes that are critical for cell proliferation, survival, adhesion and migration^21,22^. Hence, it can function in cellular signaling cascades either directly forms complex with downstream receptor protein, or regulates cellular tyrosine kinase activity, or acts as a upstream mediator for signal initiation^22^. CRKL deregulation was also proved to be involved in the EMT, progression, development, invasion, metastasis and apoptosis of a variety of cancers^21,23^. Our previous study found that CRKL was associated with proliferation, migration and invasion of murine hepatocarcinoma Hca-P^22^ and leukemia K562 cells (unpublished). CRKL has been reported to be a potential target of miR-429^24^, miR-429 could reduce CRKL protein expression in breast cancer MDA-MB-231 cells^25^, further study found miR-429 directly targeting to *CRKL*-3′-UTR in cervical cancer cells^26^. However, the exact role and underlying molecular mechanism of miR-429-CRKL axis in HCC is still unknown.

In our study, we demonstrated that miR-429 negatively regulated CRKL expression *via* selectively binding to *CRKL*-3′-UTR at 3728–3735 bp site by post-transcriptionally mediating its functionality; Furthermore, innovatively adopting miR-429 regulated HepG2 cells migration and invasion by targeting CRKL *via* inhibiting Raf/MEK/ERK-EMT pathway; Moreover, the inhibitory effect of miR-429 on HepG2 cells proliferation through the CRKL/c-Jun pathway is not powerful. The newly identified miR-429-CRKL axis partially elucidates the molecular mechanism of HCC metastasis and represents a new potential therapeutic target for HCC treatment, miR-429 overexpression in combination with CRKL knockdown may be an effective strategy in reducing HCC metastasis.

## Results

### miR-429 negatively regulates CRKL expression by selectively targeting its 3′-UTR

**Figure 1:**
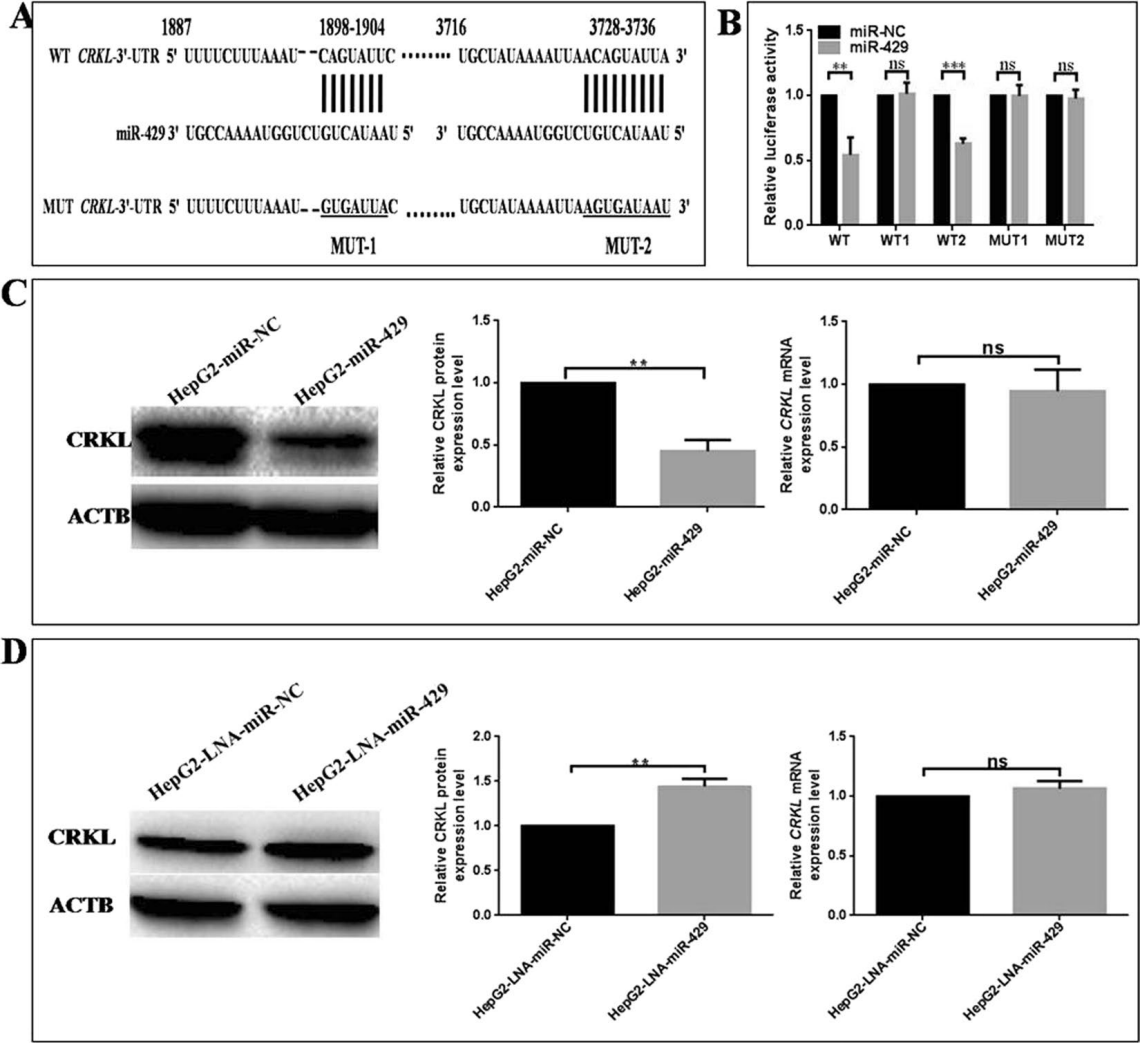
miR-429 translationally regulated CRKL expression by targeting its 3′-UTR selectively. (**A**) Putative binding sites for miR-429 at *CRKL*-3′-UTR. (**B**) Luciferase activity assay of the interaction between miR-429 and *CRKL*-3′-UTR. HepG2 cells were co-transfected with miR-429 or miR-NC and psiCHECK-2-CRKL-3′-UTR-WT or psiCHECK-2-CRKL-3′-UTR-MUT for 48 h. Renilla luciferase activity was normalized to Firefly luciferase activity. **P < 0.01, ***P < 0.001 refer to the differences were significant compared with negative control. (**C**,**D**) miR-429 negatively regulated endogenous CRKL at protein level rather than at mRNA level. The endogenous CRKL protein and mRNA levels were measured by WB and qRT-PCR in HepG2 cells transfected with miR-429, miR-NC, LNA-miR-429, LNA-miR-NC for 48 and 72 h, respectively. ACTB was used as the internal reference. **P < 0.01 refers to difference obtained in comparison with the negative control.

Bioinformatics analysis using TargetScan (http://genes.mit.edu/targetscan), PicTar (http://pictar.bio.nqyu.edu) and miRanda (http://microrna.sanger.ac.uk) tools indicated that *CRKL* was a potential target gene of miR-429, with two putative binding sites of 1898–1904 and 3728–3735 at *CRKL*-3′-UTR region for miR-429 (Fig. 1A). Luciferase reporter assay was performed to investigate *in vitro* interaction between the miR-429 and *CRKL*-3′-UTR in HepG2 cells. The miR-429 two binding sites in the *CRKL*-3′-UTR were cloned downstream of Firefly luciferase gene, the luciferase activity was decreased by 45.7 ± 1.1% (*P* = 0.004) or 37.0 ± 0.9% (*P* = 0.0008) in HepG2 cells co-transfected with the psiCHECK-2-CRKL-3′-UTR-WT or psiCHECK-2-CRKL-3′-UTR-WT2 luciferase reporter and miR-429 mimic compared with negative control cells, respectively (Fig. 1B). The relative luciferase activity was not changed in HepG2 cells co-transfected with the psiCHECK-2-CRKL-3′-UTR-WT1 and miR-429 mimic compared with negative control cells (Fig. 1B). Moreover, the inhibitory effect was abolished when the binding sites in *CRKL*-3′-UTR were mutated (Fig. 1B). The above results indicated that miR-429 directly targeting to *CRKL*-3′-UTR at 3728–3735 bp site.

Consequently, miR-429 overexpression and miR-429 supression decreased endogenous CRKL level by 55.0 ± 1.5% (*P* = 0.0089, Fig. 1C) and increased endogenous CRKL level by 44.3 ± 1.0% (*P* = 0.0038, Fig. 1D) in HepG2 cells, respectively. While interestingly, miR-429 dysexpression showed no effect on the endogenous expression level of *CRKL mRNA* in HepG2 cells (Fig. 1C,D). These results suggested CRKL was a direct downstream target of miR-429 *via* direct binding to site 2 in its 3′-UTR by post-transcriptionally mediating its functionality.

### miR-429 expression is inversely correlated with CRKL expression

**Figure 2:**
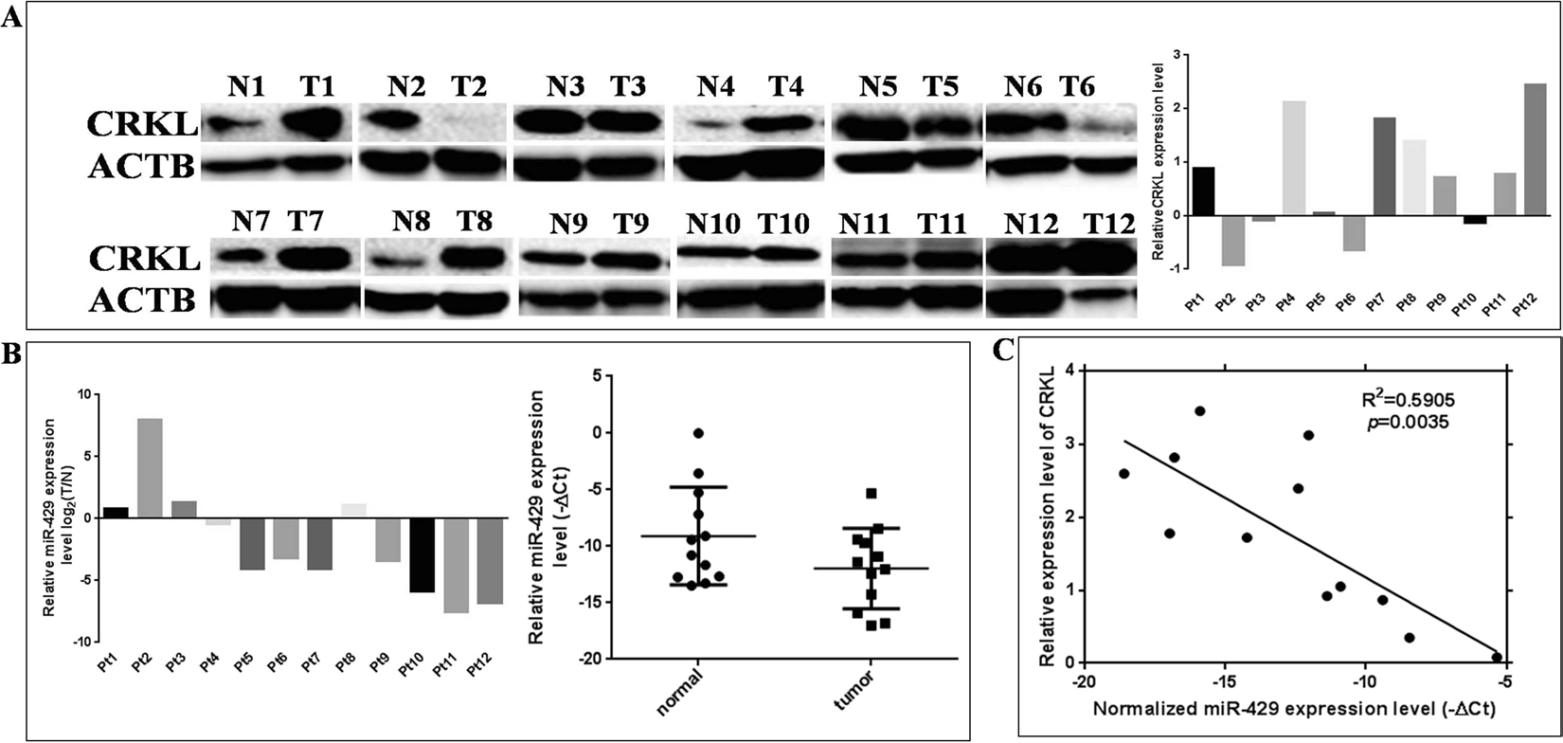
The miR-429 expression is inversely correlated with CRKL protein expression. (**A**) WB measured the protein expression level of CRKL in 12 pairs HCC and coprresponding non-tumor liver tissues. ACTB was used as the internal reference. (**B**) qRT-PCR detected miR-429 expression level in 12 pairs HCC and coprresponding non-tumor liver tissues using U6 as internal reference. (**C**) An inverse relationship between miR-429 expression and CRKL protein level was suggested in HCC tissues. R^2^ = 0.5905 with a significant *P* = 0.00035 by Spearman correlation.

To further confirm CRKL is a target of miR-429, we further detected the endogenous expression levels of miR-429 and CRKL in 12 paris of matched HCC and corresponding nontumor liver tissues. WB results showed that CRKL protein expression levels were significantly higher in HCC tissues than in corresponding nontumor liver tissues (66.7%, *P* = 0.0362, Fig. 2A). Moreover, qRT-PCR results showed that miR-429 expression levels were significantly lower in HCC tissues than that in corresponding nontumor liver tissues (*P* = 0.0295, Fig. 2B). Meanwhile, we also observed a highly significant negative correlation between the expression level of miR-429 and CRKL (R^2^ = 0.5905, *P* = 0.0035, Fig. 2C). Our results further demonstrated that miR-429 expression is negatively correlated with CRKL expression.

### miR-429 deregulation affects the *in vitro* migration and invasion of HepG2

**Figure 3:**
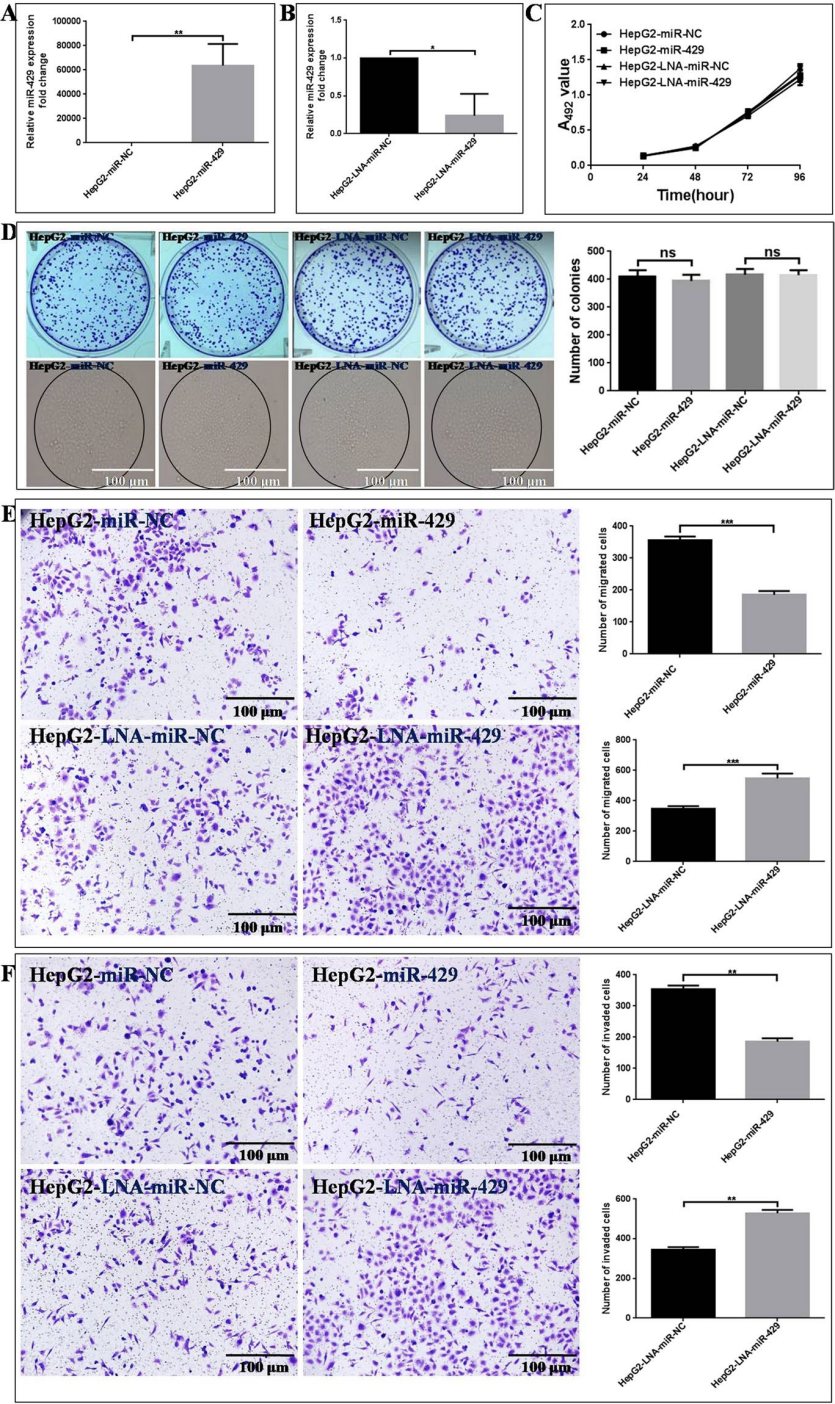
The influence of miR-429 level on the *in vitro* proliferation, colony forming, migration and invasion abilities of HepG2. (**A**) miR-429 was over-expressed in HepG2. miR-429 mimic or miR-NC were transiently transfected into HepG2 cells for 48 h, the miR-429 expression level was determined by qRT-PCR using U6 as internal reference. (**B**) miR-429 was downregulated in HepG2 cells. LNA-miR-429 or LNA-miR-NC were transiently transfected into HepG2 cells for 48 h. miR-429 level was determined by qRT-PCR using U6 as internal reference. (**C**) miR-429 level change shows no influence on HepG2 proliferation by MTT assay. (**D**) miR-429 level change exhibits no influence on HepG2 colony forming ability by clonogenic assay. (**E**,**F**) The effects of miR-429 level change on the migration and invasion capacities of HepG2 cells by Boyden transwell-chamber assay. For all the experimental results, the differences marked with *** and *** were with the *P* values below 0.05, 0.01 and 0.001 in comparison with the negative control, and ns refers to non stastical significance.

The effects of miR-429 expression level change on the proliferation, colony forming, migration and invasion properties of HepG2 cells were measured by both down-regulating and up-regulating miR-429 in HepG2. A synthetic double-stranded miR-429 mimic and single-stranded LNA-miR-429 were transiently transfected into HepG2 cells to increase and decrease miR-429 expression. qRT-PCR showed miR-429 expression level was increased by 6373600 ± 10220% (*P* = 0.0034) in HepG2-miR-429 cells compared with HepG2-miR-NC (Fig. 3A) and decreased by 76.2 ± 16.7% (*P* = 0.0103) in HepG2-LNA-miR-429 cells compared with HepG2-LNA-miR-NC cells (Fig. 3B). miR-429 deregulation showed no influence on the proliferation ability determined by MTT (Fig. 3C) and colony formation ability determined by plate colony forming assay (Fig. 3D) of HepG2 cells. Our results indicated that miR-429 was uncritical for HepG2 growth.

The deregulation of miR-429 apparently affects the *in vitro* migration and invasion capacities of HepG2 cells (Fig. 3E,F). miR-429 expression level is reversely correlated with the migration and invasion of HepG2 cells. miR-429 overexpression inhibited the migration and invasion abilities of HepG2, the numbers of migrated (185.0 ± 6.9, *P* = 0.0003, Fig. 3E) and invaded (186.0 ± 6.1, *P* = 0.0045, Fig. 3F) HepG2-miR-429 cells decreased by ~48.0% and 47.6% than HepG2-miR-NC cells (356.3 ± 6.6, 354.7 ± 6.5), respectively. Consistently, miR-429 downregulation promoted the migration and invasion abilities of HepG2 cells. The numbers of migrated (548.3 ± 17.8, *P* = 0.0006, Fig. 3E) and invaded (529.0 ± 9.5, *P* = 0.001, Fig. 3F) HepG2-LNA-miR-429 cells increased by ~57.2% and 53.3% than HepG2-LNA-miR-NC cells (348.7 ± 9.7, 345.3 ± 7.5).

### miR-429 deregulation affects the *in vitro* adhesion ability and cytoskeleton of HepG2

**Figure 4:**
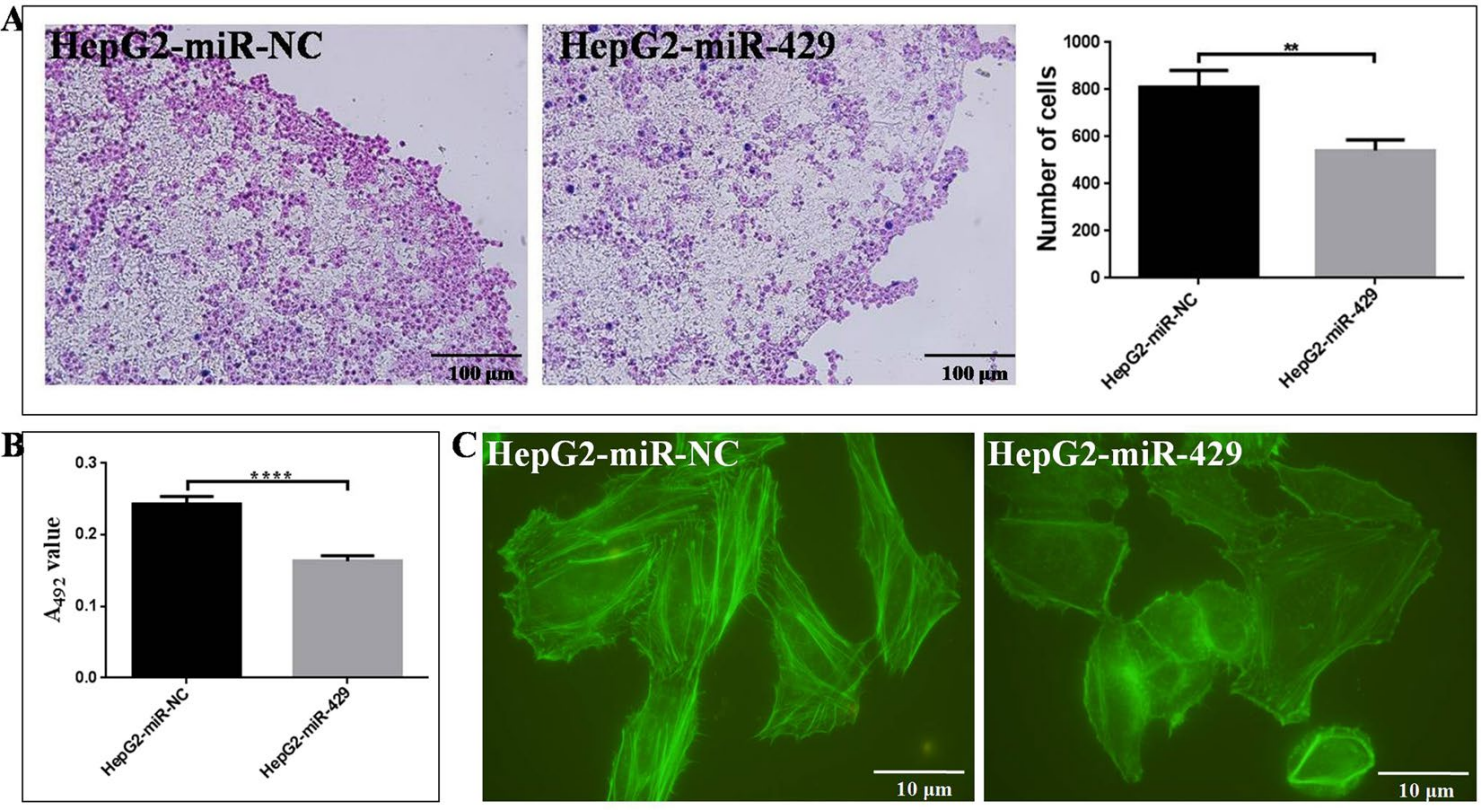
The effect of miR-429 overexpression on the cell adhesion ability and F-actin cytoskeleton expression and arrangement of HepG2. (**A**) miR-429 overexpression inhibited the *in situ* LN adhesion capacity of HepG2 (**P < 0.01). (**B**) miR-429 overexpression inhibited the extracellular matrix adhesion ability of HepG2 to FN by MTT assay (****refers to *P* value of the difference < 0.0001 than control). (**C**) miR-429 overexpression suppressed the expression and destructed the arrangement of F-actin in HepG2 cytoskeleton by FITC-phalloidin assay.

miR-429 overexpression inhibited the *in situ* lymph node (LN) adhesion potential of HepG2 cells, the numbers of HepG2-miR-429 cells (542.3 ± 25.5) adhered to LNs decreased by ~33.0% compared with HepG2-miR-NC cells (809.0 ± 41.7, *P* = 0.0005, Fig. 4A). Meanwhile, miR-429 overexpression also significantly inhibited extracellular matrix adhesion ability of HepG2 cells. HepG2-miR-429 cells showed a weaker adhesive potential to FN than HepG2-miR-NC cells. The adhesion ability of HepG2-miR-429 cells adhered to FN was decreased by ~32.8% than HepG2-miR-NC cells (*P* < 0.0001, Fig. 4B). Furthermore, miR-429 overexpression inhibited F-actin cytoskeleton protein expression of HepG2 cells. As shown in Fig. 4C, miR-429 overexpression resulted in an obvious decrease of F-actin microfilament. Cell migration and invasion is associated with actin depolymerization. Our results showed miR-429 acting as a suppressor for the HepG2 cell migration and invasion by destructing its F-actin cytoskeleton. Taken together, miR-429 overexpression inhibited the adhesion ability, cytoskeleton F-actin expression and arrangement of HepG2 cell, which leads to its decreased migration and invasion potentials.

### CRKL level positively with the *in vitro* migration and invasion of HepG2

**Figure 5:**
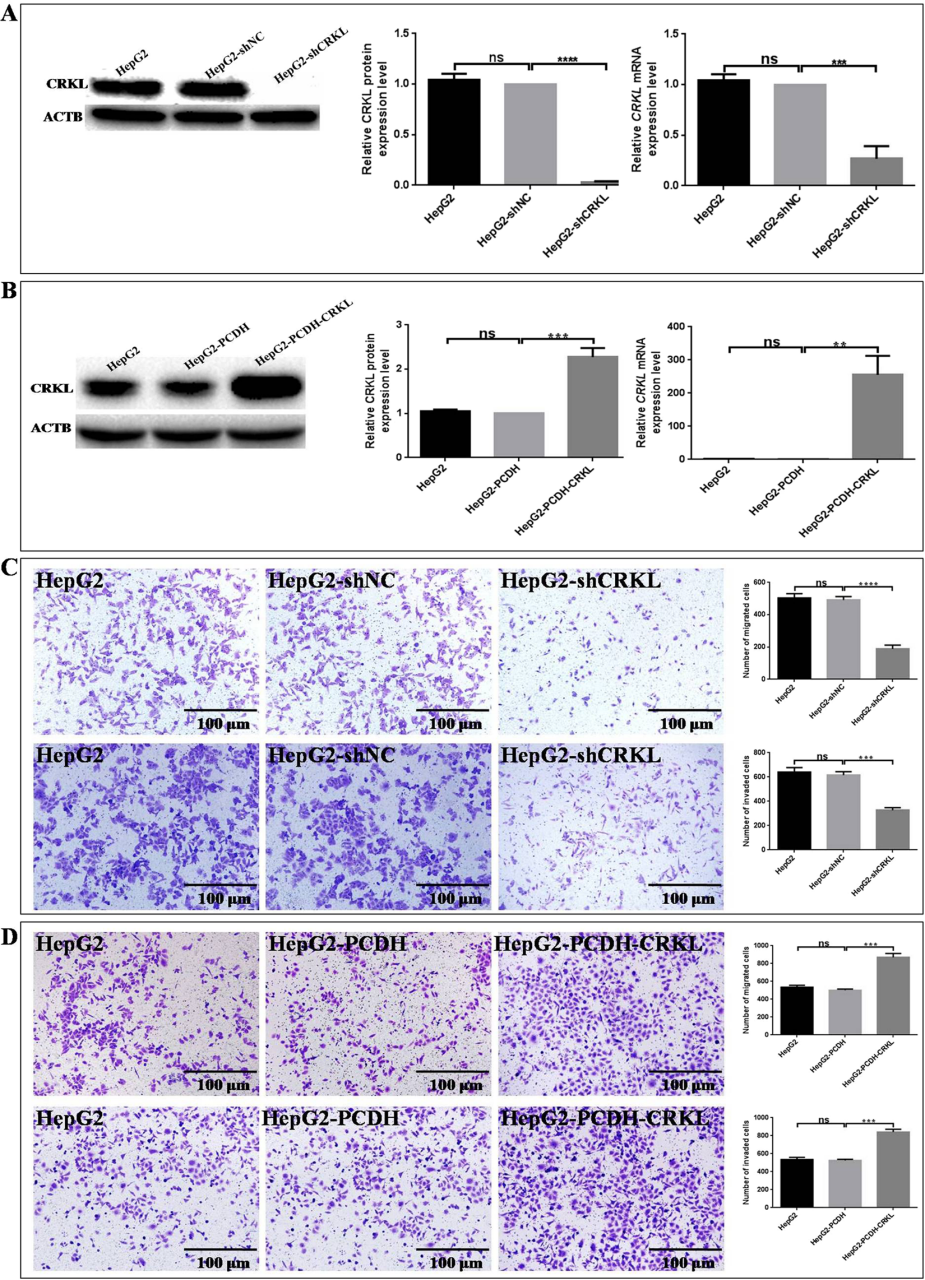
The effect of CRKL level on the *in vitro* migration and invasion abilities of HepG2 cells. (**A**) CRKL was down-regulated in HepG2. pGPU6/GFP/Neo-shRNA-CRKL and pGPU6/GFP/Neo-shRNA-NC were transfected into HepG2, selected against 400 μg/ml G418. Relative mRNA and protein levels of CRKL were determined by qRT-PCR and WB using ACTB as internal reference. *** and **** refer to the *P* values of the differences <0.001 and 0.0001, and ns refers to non stastical significance. (**B**) CRKL was up-regulated in HepG2 cells. PCDH-EF1-MCS-T2A-Puro-CRKL or PCDH-EF1-MCS-T2A-Puro were transiently transfected into HepG2 cells for 72 h. Relative mRNA and protein level was determined by qRT-PCR and WB using ACTB as internal reference. ** and *** refer to the *P* values of the differences <0.01 and 0.001, ns refers to non stastical significance. (**C**) CRKL knockdown decreases and (**D**) CRKL overexpression enhances the *in vitro* migration and invasion capacities of HepG2 cells. *** and **** refer to the *P* values of the differences <0.001 and 0.0001, ns refers to non stastical significance.

The effect of CRKL deregulation on the migration and invasion capacities of HepG2 were investigated by both down-regulating and up-regulating its cellular expression level. The monoclonal HepG2-shCRKL and HepG2-shNC cells transfected with specific shRNA of *CRKL* and unrelated targeting shRNA were obtained against G418 screening by limited dilution method. No mRNA and protein level changes were observed for CRKL between HepG2-shNC and HepG2 cells (Fig. 5A). In comparison with HepG2-shNC, WB and qRT-PCR assays indicated that CRKL protein and *CRKL* mRNA levels were decreased by 97.0 ± 0.6% (*P* < 0.0001) and 73.3 ± 7.2% (*P* = 0.0005, Fig. 5A) in HepG2-shCRKL cells. The establishment of monoclonal HepG2-shCRKL cells with stable CRKL knockdown ensured the investigation of CRKL in HCC. Meanwhile, we transiently transfected HepG2 cells with PCDH-EF1-MCS-T2A-Puro-CRKL expression plasmid to overexpress CRKL. CRKL protein and *CRKL* mRNA levels were increased by 127.3 ± 12.0% (*P* = 0.0005) and 25400 ± 3329% (*P* = 0.0016, Fig. 5B) in HepG2-PCDH-CRKL cells than in HepG2-PCDH cells, which provides control study for the upregulation effect of CRKL on HepG2 cell malignant behaviours.

CRKL level was positively correlated with the migration and invasion capacities of HepG2 cells. As shown in Fig. 5C, the numbers of migrated and invaded HepG2-shCRKL cells were measured as 188.0 ± 13.6 and 326.7 ± 11.9, which were only ~38.3% (*P* < 0.0001) and ~53.1% (*P* = 0.0001) of HepG2-shNC cells (491.3 ± 12.0, 615.0 ± 16.6). Consistently, CRKL overexpression promoted the migration and invasion abilities of HepG2. The numbers of migrated and invaded HepG2-PCDH-CRKL cells (870.0 ± 24.9, 841.0 ± 18.1) were ~1.7- (*P* = 0.0001, Fig. 5D) and 1.6-folds (*P* = 0.0001, Fig. 5D) of HepG2-PCDH cells (501.3 ± 6.9, 525.3 ± 7.2). Clearly, CRKL affects the *in vitro* migration and invasion abilities of HepG2 cells.

### CRKL positively correlates with cell adhesion ability and cytoskeleton arrangement of HepG2

**Figure 6:**
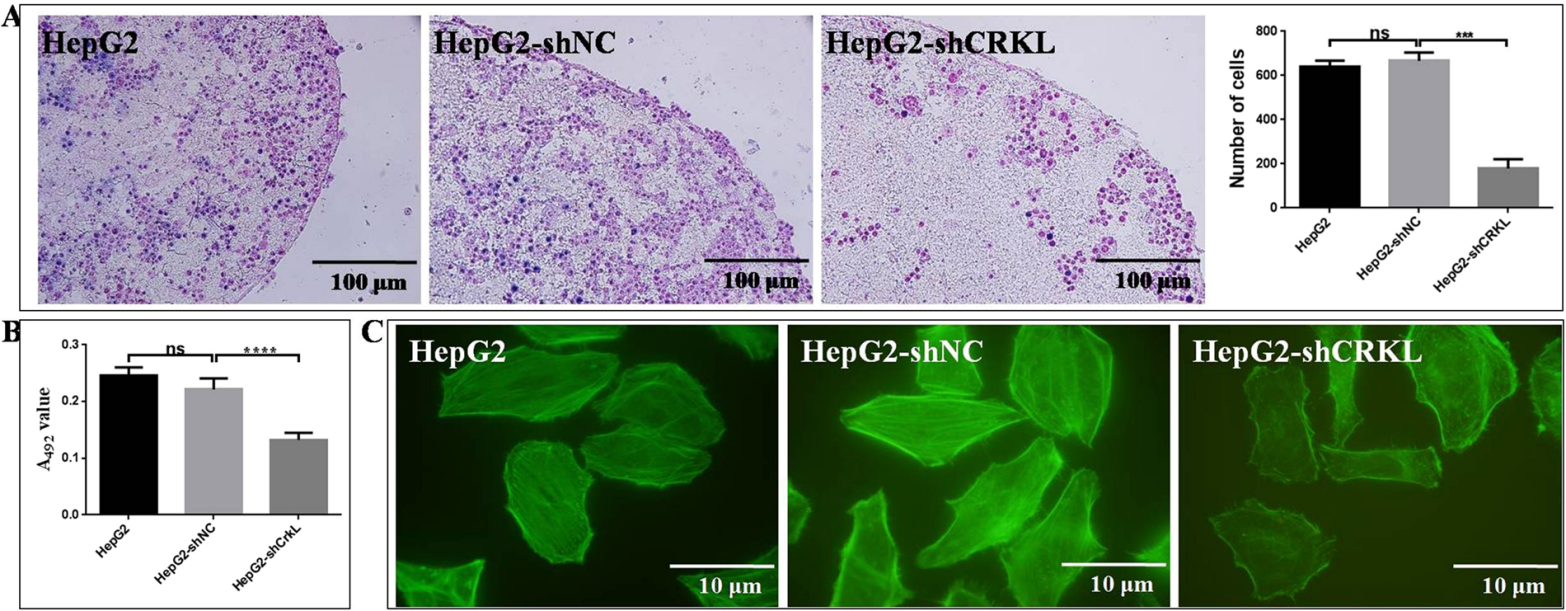
The effect of CRKL knockdown on cell adhesion ability and cytoskeleton F-actin in HepG2. (**A**) CRKL knockdown inhibited the *in situ* LN adhesion capacity of HepG2 cells (**** refers to *P* < 0.0001). (**B**) CRKL knockdown inhibited the extracellular matrix adhesion ability of HepG2 cells to FN by MTT assay (****P < 0.0001). (**C**) CRKL knockdown decreased cytoskeleton F-actin protein expression and filament arrangement of HepG2 cells by FITC-phalloidin assay.

CRKL knockdown inhibited the *in situ* lymph node (LN) adhesion potential of HepG2. The numbers of HepG2-shCRKL cells (179 ± 24.6) adhered to LNs decreased by ~73.1% than HepG2-shNC cells (666.3 ± 21.8, *P* < 0.0001, Fig. 6A). There were no difference between HepG2-shNC and HepG2 (640 ± 15.9, *P* = 0.3846, Fig. 6A) cells. Meanwhile, CRKL knockdown inhibited the extracellular matrix adhesion ability of HepG2 cells to FN. HepG2-shCRKL cells showed a much lower adhesive potential to FN than HepG2-shNC cells. The adhesion ability of HepG2-shCRKL to FN was decreased by ~40.2% than HepG2-shNC cells (*P* < 0.0001, Fig. 6B). No difference was observed between HepG2-shNC and HepG2 cells (*P* = 0.4336, Fig. 6B). Furthermore, CRKL knockdown inhibited F-actin cytoskeleton protein expression and microfilament arrangement of HepG2 cells. As shown in Fig. 6C, CRKL knockdown resulted in obviously decreased microfilament of F-actin, while, there was no difference between HepG2-shNC and HepG2 cells. CRKL knockdown inhibited the LN and FN adhesion potential, F-actin cytoskeleton protein expression and arrangement of HepG2 cells, leading to its decreased migration and invasion potential.

### CRKL promotes the proliferation and colony forming capacities of HepG2

**Figure 7:**
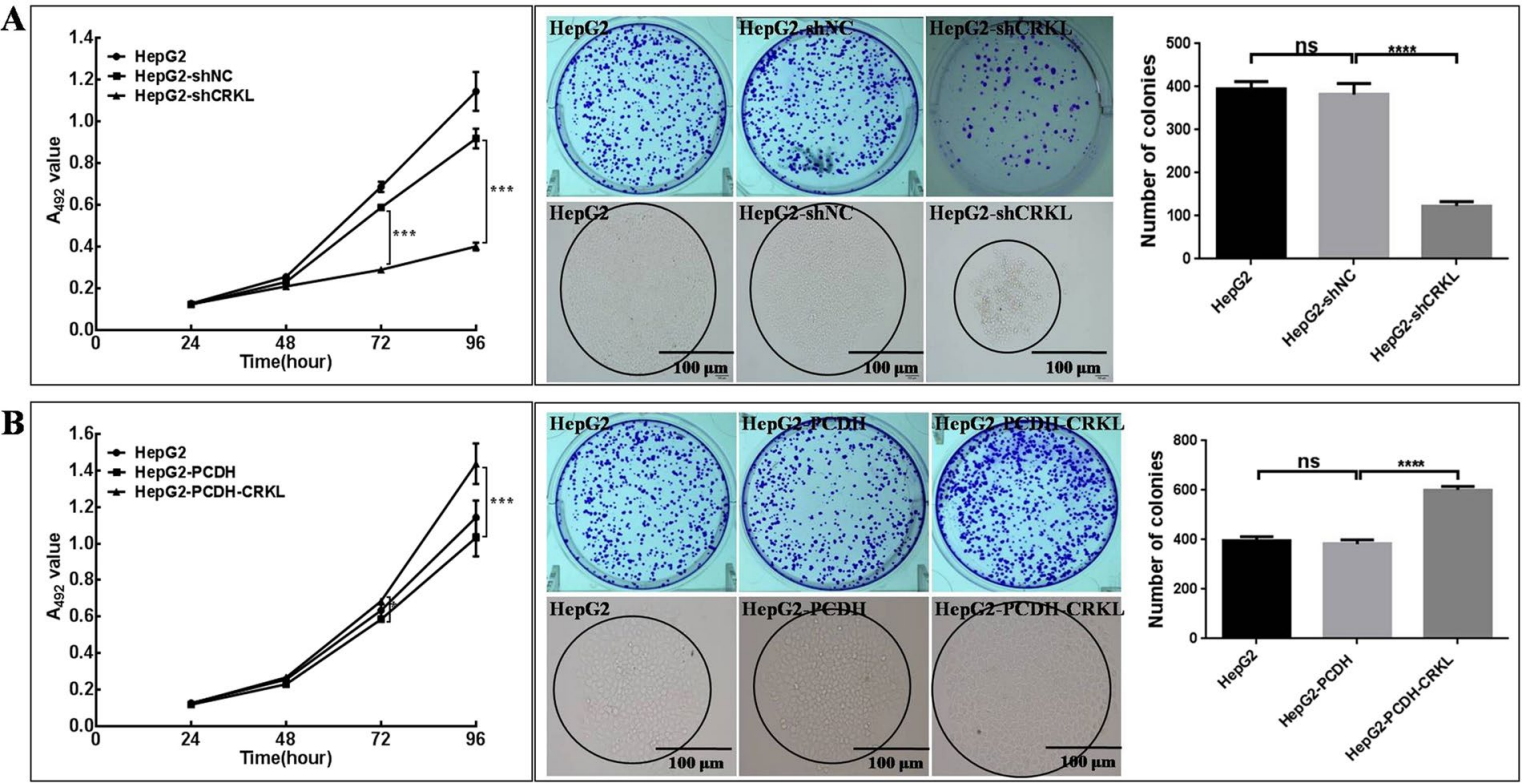
The effect of CRKL level change on HepG2 growth. (**A**) CRKL knockdown decreased the proliferation and colony formation abilities of HepG2 cells. (**B**) CRKL overexpression promoted the proliferation and colony formation ability of HepG2 cells. *** and **** refer to the differences with *P* < 0.001 and 0.0001, ns refers to no statistical difference, respectively.

CRKL level positively correlates with the *in vitro* proliferation of HepG2 cells. No proliferation difference was observed between HepG2-shNC and HepG2 cells by MTT assay. While, the stable CRKL knockdown resulted in decreased proliferation abilities by ~50.8% and 56.4% (*P* < 0.001, Fig. 7A) of HepG2-shCRKL cells at the inoculation time intervals of 72 h and 96 h than HepG2-shNC cells, respectively. Consequently, CRKL overexpression increased the proliferation of HepG2 cells. The relative proliferation capacities of HepG2-PCDH-CRKL cells were measured ~17.0% and 39.2% higher than HepG2-PCDH cells at the time intervals of 72 h (*P* < 0.05) and 96 h (*P* < 0.001, Fig. 7B). No difference was determined between HepG2-PCDH and HepG2 cells (Fig. 7B).

CRKL expression level is also positively associated with the colony forming ability of HepG2 cells. Plate colony formation assay indicated that CRKL downregulation reduced the colony forming efficiency of HepG2 cells. The number of formed colonies of HepG2-shCRKL cells was 123.3 ± 6.0 per view that was only about 32.3% of that of HepG2-shNC cells (382.0 ± 14.8, *P* < 0.0001, Fig. 7A). The colony size of HepG2-shCRKL cells was smaller than HepG2-shNC cells. Comparable colony forming capacity was detected for HepG2-shNC and HepG2 cells. Consistently, CRKL overexpression improved colony forming capacity of HepG2, the number of HepG2-PCDH-CRKL cells was 601.7 ± 7.5 per view that was 57.2% higher than that of HepG2-PCDH cells (382.7 ± 9.5, *P* < 0.0001, Fig. 7B). The size of colony of HepG2-PCDH-CRKL cell was enlarged than HepG2-PCDH due to CRKL overexpression. No difference was determined between HepG2-PCDH and HepG2 cells.

### CRKL overexpression reversed miR-429-induced inhibition on HepG2 migration and invasion

**Figure 8:**
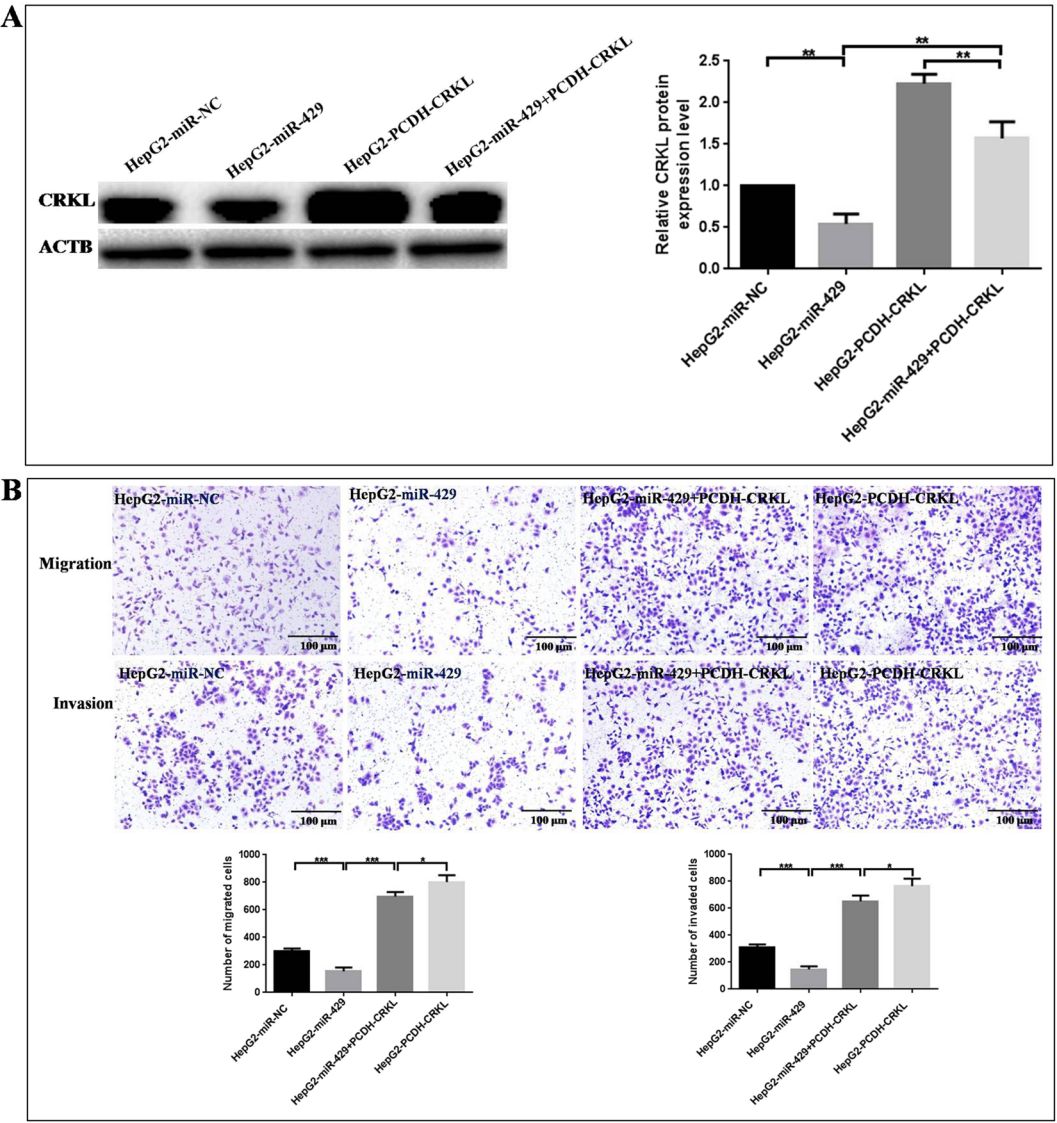
CRKL overexpression reversed miR-429-induced suppression on the *in vitro* migration and invasion of HepG2. (**A**) Expression level comparisons of CRKL in HepG2 cells transfected with miR-NC, miR-429 mimic, PCDH-CRKL and miR-429 + PCDH-CRKL. (**B**) CRKL overexpression neutralized miR-429-induced inhibition on the migration and invasion of HepG2 cells. *^,^** and *** means the difference is with a *P* value below 0.05, 0.01 and 0.001 compared with the control.

Is CRKL a direct functional mediator of miR-429-inhibited migration and invasion for HepG2 cells? Could the *in vitro* phenotypes associated with miR-429 deregulation be reversed by opposite CRKL expression? We performed a “rescue” experiment by co-tansfecting miR-429 mimic and PCDH-CRKL vectors into HepG2 cells. miR-429 could downregulate endogenous CRKL expression level in HepG2 cells, while, it could only downregulate the CRKL expression level in HepG2 cells co-transfected with miR-429 and PCDH-CRKL vector to certain extent (Fig. 8A) as PCDH-CRKL expression vector does not have 3′-UTR domain. Meanwhile, exogenous CRKL overexpression markedly counteracted the suppression effect of miR-429 on the migration and invasion capacities of HepG2 cells (Fig. 8B, *P* < 0.001). The above results proved that miR-429 inhibited HepG2 migration and invasion by downregulating CRKL through binding with its *CRKL*-3′-UTR. CRKL was a functional effector of miR-429 mediated anti-tumor effect.

### miR-429-CRKL axis mediates tumor cell migration and invasion *via* Raf/MEK/ERK and EMT

The underlying molecular mechanism of miR-429-CRKL axis in HCC is unknown. Current work linked miR-429-mediated action on biological behaviours of HepG2 *via* Raf/MEK/ERK and EMT by targeting CRKL.

**Figure 9:**
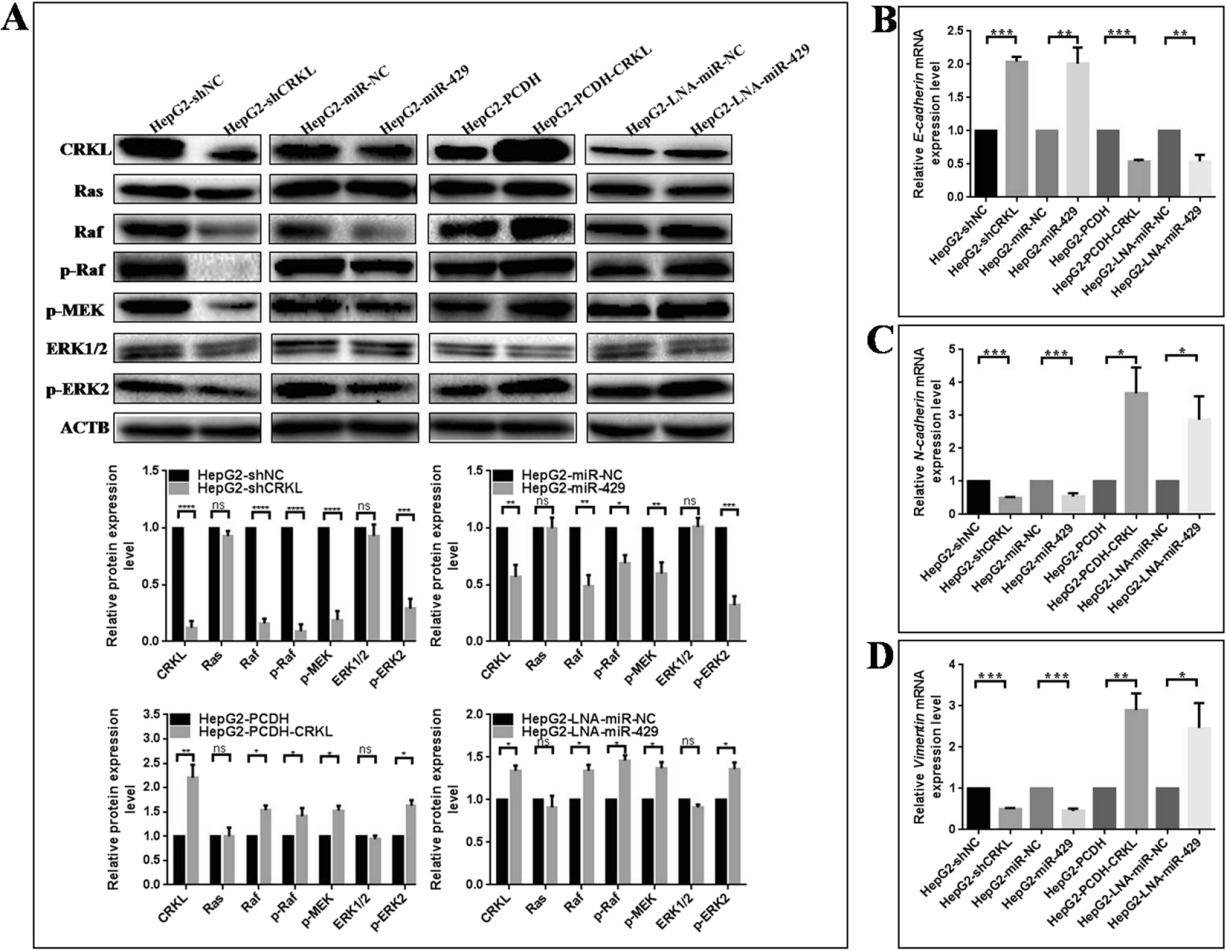
The influences of miR-429 and CRKL on Raf/MEK/ERK pathway and EMT in HepG2 cells. (**A**) Comparative analysis of Ras, Raf, p-Raf, p-MEK, ERK1/2 and p-ERK2 protein levels in HepG2 cells transfected with miR-429 mimic, miR-429 inhibitor, shCRKL or PCDH-CRKL. (**B**–**E**) Comparative analysis of mRNA levels of *E-cadherin, N-cadherin, Vimentin* and *c-Jun* in above group HepG2 cells. *^,^** and *** refer to the level changes were in statistical significances with *P* < 0.05, 0.01 and 0.001, respectively, ns refers to no statistical difference.

We found CRKL knockdown or miR-429 overexpression resulted in the similar trend on downregulating the protein expression levels of Raf, p-Raf, p-MEK and p-ERK2 in HepG2 cells (Fig. 9A). Consistently, CRKL overexpression or miR-429 silencing increased the protein expression levels of Raf, p-Raf, p-MEK and p-ERK2. No changes were observed for Ras and ERK1/2 (Fig. 9A). Clearly, miR-429-CRKL axis mediates the biological properties of HepG2 *via* Raf/MEK/ERK signaling pathway.

**Figure 10:**
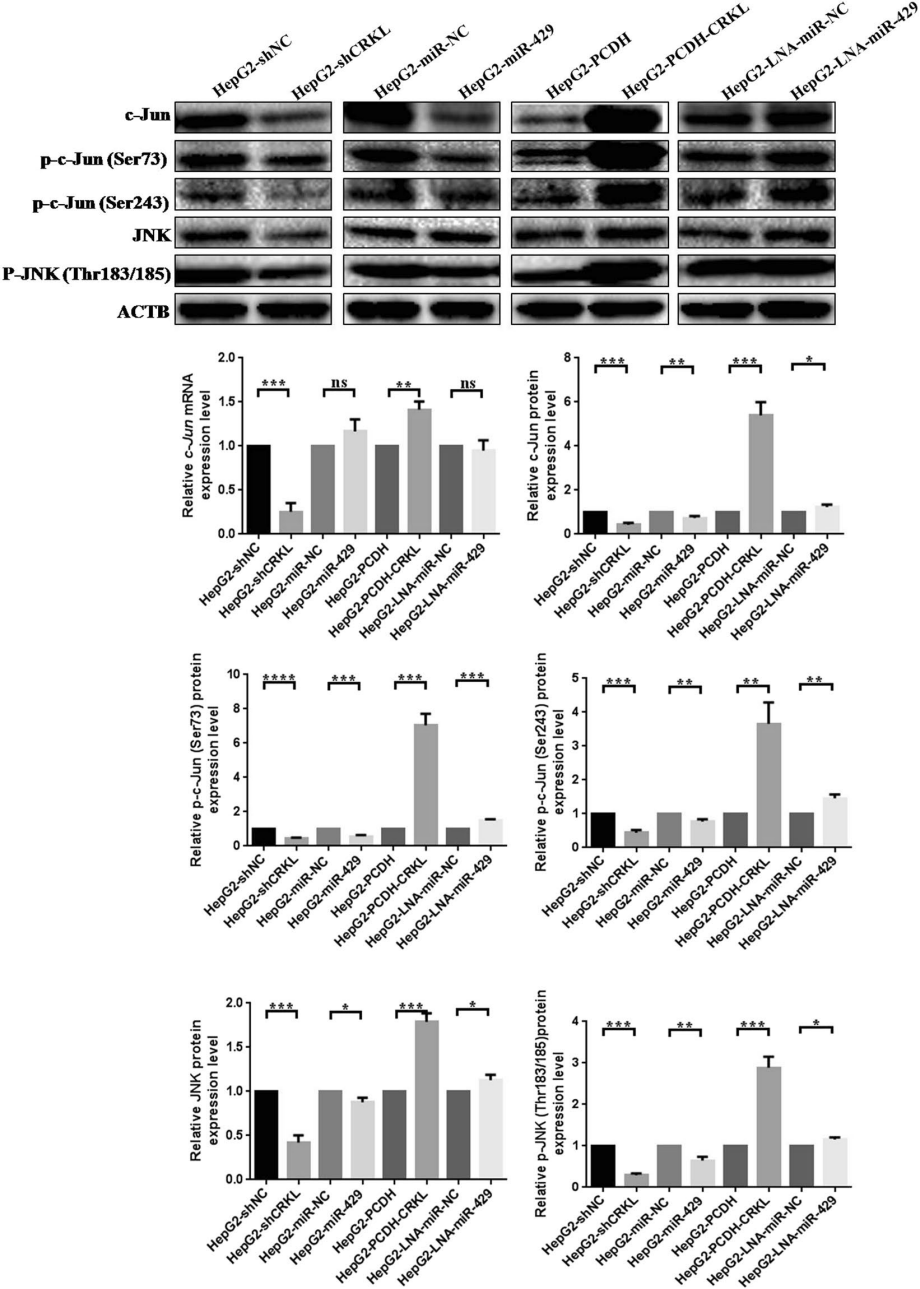
The influences of miR-429 and CRKL on c-Jun pathway in HepG2 cells. WB detected the protein expression level of c-Jun, p-c-Jun (73), p-c-Jun (243), JNK, p-JNK in HepG2 cells transfected with miR-429 mimic, miR-429 inhibitor, shCRKL or PCDH-CRKL. qRT-PCR also detected the mRNA expression level of c-Jun in HepG2 cells transfected with miR-429 mimic, miR-429 inhibitor, shCRKL or PCDH-CRKL. *^,^**^,^ *** and **** refer to the level changes were in statistical significances with *P* < 0.05, 0.01, 0.001 and 0.0001, respectively, ns refers to no statistical difference.

miR-429-CRKL axis influence the EMT property of HepG2 cells. We examined the expression level changes of EMT markers, *E-cadherin*, *N-cadherin* and *Vimentin* in HepG2 cells following CRKL or miR-429 dysexpression. qRT-PCR assay showed CRKL knockdown or miR-429 overexpression increased the expression level of epithelial marker *E-cadherin* (Fig. 9B), and decreased the expression levels of mesenchymal marker *N-cadherin* (Fig. 9C) and *Vimentin* (Fig. 9D). CRKL overexpression or miR-429 silencing decreased *E-cadherin* expression (Fig. 9B), and increased *N-cadherin* (Fig. 9C) and *Vimentin* expression (Fig. 9D). miR-429 and CRKL are involved in the EMT of HCC.

c-Jun signaling pathway is critical for cell growth. CRKL knockdown decreased and overexpression increased the mRNA level of *c-Jun*, while miR-429 overexpression and silencing showed no influence on *c-Jun* expression (Fig. 10). Interestingly, CRKL knockdown or overexpression significantly decreased or increased the protein expression levels of c-Jun, p-c-Jun (73), p-c-Jun (243), JNK, p-JNK by 57.7%, 56.1%, 45.2%, 3.6-fold or 4.4-fold, 6.0-fold, 3.6-fold, 78.7%, 1.9-fold (Fig. 10). While, miR-429 overexpression or silencing only slightly decreased or increased the protein levels of c-Jun, p-c-Jun (73), p-c-Jun (243), JNK, p-JNK by 26.1%, 43.2%, 27.5%, 12.1%, 35.1% or 23.0%, 49.2%, 52.3%, 12.7%, 15.9% (Fig. 10). Our results showed that the deregulate degree of miR-429 on c-Jun pathway was significantly weaker than CRKL, this difference might contribute to explain our previous results that the overexpression or silencing of miR-429 rarely influenced the proliferation and colony formation abilities of HepG2 cells, while CRKL dysexpression significantly interrupted the proliferation and colony forming abilities of HepG2 cells, indicating the deregulate degree of miR-429 on c-Jun pathway was not enough to regulate the proliferation and colony formation abilities of HepG2 cells. Our results also demonstrated that miR-429-CRKL axis regulates c-Jun pathway at posttranscriptional level in HepG2 cells.

**Figure 11:**
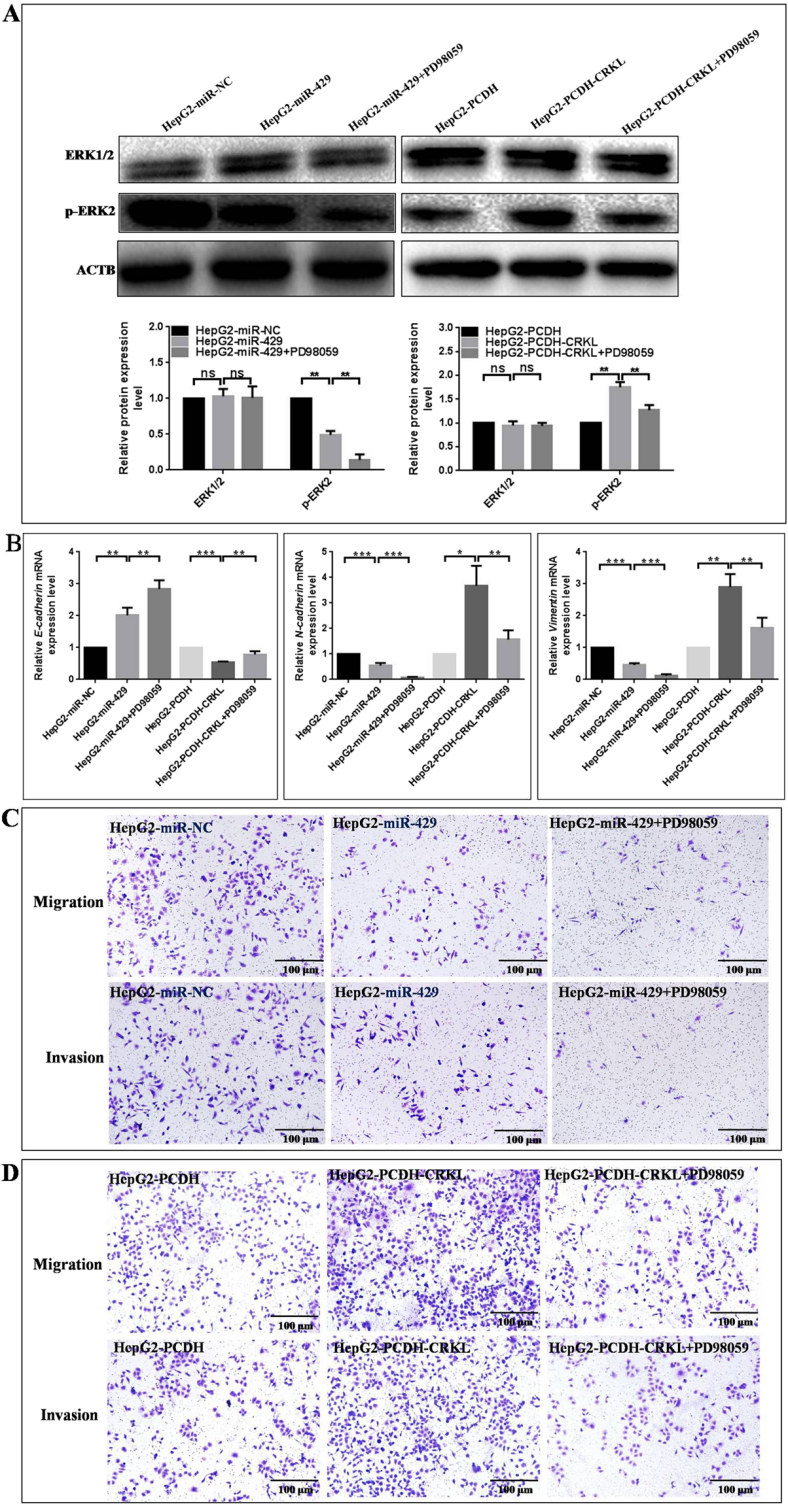
PD98059 influence on miR-429- and CRKL-induced Raf/MEK/ERK pathway and EMT. (**A**) WB assay of ERK and p-ERK2 level changes in HepG2-miR-429 and HepG2-PCDH-CRKL cells with PD98059 treatment for 48 h. (**B**) qRT-PCR assay of *E-cadherin, N-cadherin, Vimentin* changes in HepG2-miR-429 and HepG2-PCDH-CRKL cells with PD98059 treatment for 48 h. The influences of PD98059 on migration and invasion abilities of HepG2-miR-429 (**C**) and HepG2-PCDH-CRKL cells (**D**) *** and *** refer to the level changes were in statistical significances with *P* < 0.05, 0.01 and 0.001, respectively, ns refers to no statistical difference.

The linkages of Raf/MEK/ERK pathway and EMT to miR-429- and/or CRKL-mediated tumor migration and invasion were further validated using PD98059, a specific pERK1/2 inhibitor, for specific signaling blocking. The treatment of HepG2-miR-429 and HepG2-PCDH-CRKL cells with 20 μg/ml PD98059 resulted in reduced protein expression of pERK2 (Fig. 11A), reduced mRNA levels of *N-cadherin* and *Vimentin*, and increased mRNA level of *E-cadherin* (Fig. 11B). As in Fig. 11C, miR-429 overexpression significantly inhibited the migration and invasion potentials of HepG2 cells, which were further enhanced in the presence of PD98059. The presence of PD98059 tremendously reversed the promotion of CRKL overexpression on the migration and invasion potentials of HepG2 cells (Fig. 11D). These results concluded miR-429 and CRKL regulates HepG2 cell migration and invasion *via* Raf/MEK/ERK-EMT pathway, which also revealed the mutual influence relationship between miR-429 and CRKL, providing a novel insight for investigating the combined power of miR-429 and CRKL in HCC field.

## Discussion

miRNAs are attractive candidates for human malignancies^27^. Considerable attention is globally attracted in understanding the role of miRNAs in the tumorigenesis, diagnosis, classification and prognosis of a variety of cancers^28,29^. miR-200 family including miR-200a, miR-200b, miR-200c, miR-429 and miR-141, played important roles in development, progression, metatstasis and chemoresistance of tumors^12,18,30,31^. We previously debected the effect of miR-200a, miR-200b, miR-200c, miR-429 and miR-141 on CRKL expression by up-regulating miR-200a, miR-200b, miR-200c, miR-429 and miR-141 in HepG2 cells, respectively, our results showed that miR-429 and miR-141 significantly inhibited endogenous CRKL expression level compared to miR-200a, miR-200b, miR-200c, however, miR-141 have no affect on migration and invasion abilities of HepG2 cells, so we selected miR-429 to investigate its function and mechanism in HepG2 cells.

miR-429 abnormal expression has been linked to osteosarcoma, renal cancer, ovarian cancer, glioma, breast cancer, oral squamous cell carcinoma, gastric cancer, esophagus cancer, cervical cancer, bladder cancer, lung cancer, prostate cancer, colon cancer and liver cancer^30^. It shows suppression or promotion effects on the tumor development, invasion, metastasis, apoptosis and drug-resistance^31^ depending on the tumor type and subtype. It is a potential indicator for the diagnosis, treatment and prognosis of certain tumors.

As a mutli-functional adaptor protein in signal transduction, CRKL misregulation is involved in a variety of cancers^21^. It is an attractive target for diagnostics, treatment and prognosis of certain cancers^22,32^. Previously reported that CRKL was a potential target gene of miR-429, miR-429 reduced CRKL protein expression in breast cancer MDA-MB-231 cells^25^, further study found miR-429 directly targeting to *CRKL*-3′-UTR by luciferase reporter assay in cervical cancer cells^26^, indicating CRKL was a direct target gene of miR-429. However, the exact role and underlying molecular mechanism of miR-429-CRKL axis in HCC is still unknown. In our study, we reported miR-429 directly binding to site 2 in *CRKL*-3′-UTR regulated HCC cell migration and invasion *via* Raf/MEK/ERK-EMT pathway.

We found CRKL with two putative binding sites of 1898–1904 and 3728–3735 at *CRKL*-3′-UTR region for miR-429 by bioinformatics analysis, our results showed miR-429 directly targeting to *CRKL*-3′-UTR at 3728–3735 bp site (Fig. 1B), which was consistent with previous report^33^. miR-429 could decrease CRKL protein expression level in breast cancer^25^ and cervical cancer cells^26^, consistently, our results showed miR-429 overexpression decreased endogenous CRKL protein expression level (Fig. 1C) and miR-429 silencing increased endogenous CRKL protein expression level (Fig. 1D) in HepG2 cells. However, the effect of miR-429 deregulation on *CRKL* mRNA expression level is still unknown, we found miR-429 dysexpression showed no effect on endogenous *CRKL* mRNA expression level in HepG2 cells (Fig. 1C,D). The results suggested CRKL was a direct downstream target of miR-429 *via* direct binding to site 2 in its 3′-UTR by post-transcriptionally mediating its functionality. Our “Rescue” experiments indicated miR-429 could not downregulate exogenous CRKL expression on account of PCDH-CRKL without 3′-UTR, which further verified miR-429 directly targeting *CRKL*-3′-UTR to downregulate its expression. Our results demonstrated miR-429 could negatively regulate CRKL expression *in vitro*, we also further demonstrated CRKL protein expression inversely correlates with miR-429 expression in HCC tissues (Fig. 2).

Previous studies have reported that CRKL could affect cancer cell proliferation^21^. In our study, CRKL also indeed affected HepG2 cells proliferation and colony abilities, CRKL knockdown significantly inhibited HepG2 cells proliferation and colony abilities (Fig. 7A), while, CRKL overexpression promoted HepG2 cells proliferation and colony abilities (Fig. 7B). However, re-expression or silencing of miR-429 cannot affect HCC cells proliferation (Fig. 3C) and colony formation abilities (Fig. 3D). CRKL was only downregulated or upregulated about 55% (Fig. 1C) and 44% (Fig. 1D) by miR-429 mimics or inhibitors, respectively, while, CRKL downregulated or upregulated about 97% or 127% in our obtained monoclonal HepG2-shCRKL or transiently transfected HepG2-PCDH-CRKL cells, we speculate the underlying cause of this phenomenon is the deregulate degree of CRKL expression level was not enough, indicating CRKL only deregulated to a certain degree, which can affect HepG2 cells proliferation ability. We further found that CRKL knockdown or overexpression significantly decreased or increased the protein levels of c-Jun, p-c-Jun (73), p-c-Jun (243), JNK, p-JNK, while, miR-429 overexpression or silencing only slightly decreased or increased the protein levels of c-Jun, p-c-Jun (73), p-c-Jun (243), JNK, p-JNK (Fig. 10). We think the deregulate degree of CRKL by miR-429 was not enough to significantly affected on c-Jun pathway, so the deregulate degree of miR-429 on c-Jun pathway was significantly weaker than CRKL, indicating the deregulate degree of miR-429 on c-Jun pathway was not enough to regulate the proliferation and colony formation abilities of HepG2 cells. Although miR-429 was confirmed to target CRKL directly, we think that the inhibitory effect of miR-429 on HepG2 cells proliferation through the CRKL/c-Jun pathway is not powerful.

In contrast, CRKL contributed to miR-429-mediated metastasis inhibition. We demonstrated that re-expression of miR-429 was remarkably effective in suppressing HepG2 cells migration and invasion *in vitro* (Fig. 3E), in addition, silencing of miR-429 was significantly promoted HepG2 cells migration and invasion abilities (Fig. 3F). Furthermore, CRKL knockdown or overexpression exhibited similar effects as the overexpression or silencing of miR-429 (Fig. 3C,D). Adhesion is an important step in tumor metastasis, miR-429 overespression inhibited the *in situ* lymph node and extracellular matrix FN adhesion potentials (Fig. 4), consistently, CRKL knockdown also inhibited the *in situ* lymph node and extracellular matrix FN adhesion potential (Fig. 6). F-actin depolymerization is associated with cell movement, our results showed miR-429 overexpression resulted in an obvious decrease of F-actin microfilament (Fig. 4), meanwhile, CRKL knockdown also resulted in obviously decreased of F-actin microfilament (Fig. 6). Our results demonstrated miR-429-CRKL axis affected HepG2 cell migration and invasion potentials by regulating the adhesion ability, cytoskeleton F-actin expression and arrangement of HepG2 cell.

Are the miR-429-mediated influences on HepG2 properties linked to CRKL dysexpression? Is CRKL a direct functional mediator of miR-429 on HepG2 cells migration, invasion and associated *in vitro* phenotypes? The “rescue” experiment found miR-429 could not downregulate exogenous CRKL expression on account of PCDH-CRKL without 3′-UTR (Fig. 8A). Meanwhile, CRKL overexprssion counteracted the inhibition effect of miR-429 on HepG2 cells migration and invasion (Fig. 8B). The above results once again proved that CRKL is a functionally relevant effector of miR-429 mediated antimetastatic effect, and miR-429 inhibited HepG2 cells migration and invasion only by directly targeting *CRKL*-3′-UTR to downregulate its expression.

miR-429-CRKL axis, a new antimetastatic regulator for HCC shown to significantly suppress HCC invasion and metastasis *in vitro* and it maybe a novel potential therapeutic target for HCC treatment. These findings open a novel avenue to investigate the molecular mechanism of HCC progression and to develop potential therapeutics against HCC. However, no studies have reported a definitive mechanism for miR-429-CRKL axis in HCCs. Our results showed that miR-429 and CRKL affected the expression level of Raf/MEK/ERK pathway- and EMT-related molecules (Fig. 9), we speculated miR-429-CRKL axis might mediate tumor migration and invasion *via* regulating Raf/MEK/ERK-EMT pathway. We validated the potential involvement of Raf/MEK/ERK-EMT using a specific ERK inhibitor PD98059 (Fig. 11A), the expression level of *E-cadherin* increased and *N-cadherin, Vimentin* decreased after blocking Raf/MEK/ERK pathway by ERK inhibitor PD98059 (Fig. 11B), indicating Raf/MEK/ERK pathway could regulate EMT. Furthermore, PD98059 exacerbated the effect of miR-429 overexpression on HepG2 cells migration and invasion abilities (Fig. 11C), PD98059 weakened the effect of CRKL overexpression on HepG2 cells migration and invasion abilities (Fig. 11D). The results indicated that miR-429-CRKL axis regulated HepG2 cell migration and invasion *via* Raf/MEK/ERK-EMT pathway.

Current work showed the deregulation of CRKL or miR-429 could induce both the expression level changes of Raf and p-Raf in HepG2 cells. In fact, except for the involvement of expression level change of p-Raf in a canonical regulation fashion in diseases, the differential expression of total Raf in human tumors or in cancer cells induced by other molecule has commonly been regarded as signal transduction change from Raf/MEK/ERK. In a lot of published papers, if the up-stream molecules and down-stream molecules for Raf or p-Raf were found deregulated in the detection systems, the authors commonly declared that investigated genes, proteins, miRNA or drugs worked through Raf pathway^34–37^. Raf is the key point in the Raf/MEK/ERK pathway, the Raf family consists of three isoforms: A-Raf, B-Raf, and Raf-1, much of the unprecedented research involving Raf was carried out on Raf-1^38^. It was reported that the overexpression of Raf-1 was significantly associated with a shorter DFS (disease free survival) and a poor OS (overall survival) of HCC patients as an independent risk factor for HCC recurrence and death^36^. These reports were consistent with our results obtained from current work, and CRKL-deregulation associated with expression changes of Raf and p-Raf in CML patients’ specimens and K562 cells (unpublished) as well as in ccRCC patients’ tissues and 786-O and ACHN cells (unpublished). In gastric cancer (GC), by targeting SLC34A2, the overexpression of miR-939 in GC cells significantly decreased Raf-1, p-MEK1/2 and p-ERK expression levels for suppressing the chemoresistance and metastasis of GC^34^. MiR-7 was reported to be able to inhibit glioblastoma growth by simultaneously interfering with the PI3K/ATK and Raf/MEK/ERK pathways. MiR-7 markedly decreased the expression of Raf-1^39^. Moreover, compared with normal tissues, Raf-1, ERK and p-ERK were overexpressed in malignant breast tumor tissues. MiR-195 and miR-497 inhibited corresponding breast cancer cells proliferation and invasion *via* decreasing Raf-1, ERK, p-ERK expression levels^35^. Taken together, our results highly implicated that CKRL or miR-429 deregulation affected Raf/MEK/ERK signal transduction in mediating the metastatical behaviours of HepG2. Current work provides certain new clue to the regulation mechanism for miR-429-CRKL functionality in tumorigenicity.

**Figure 12:**
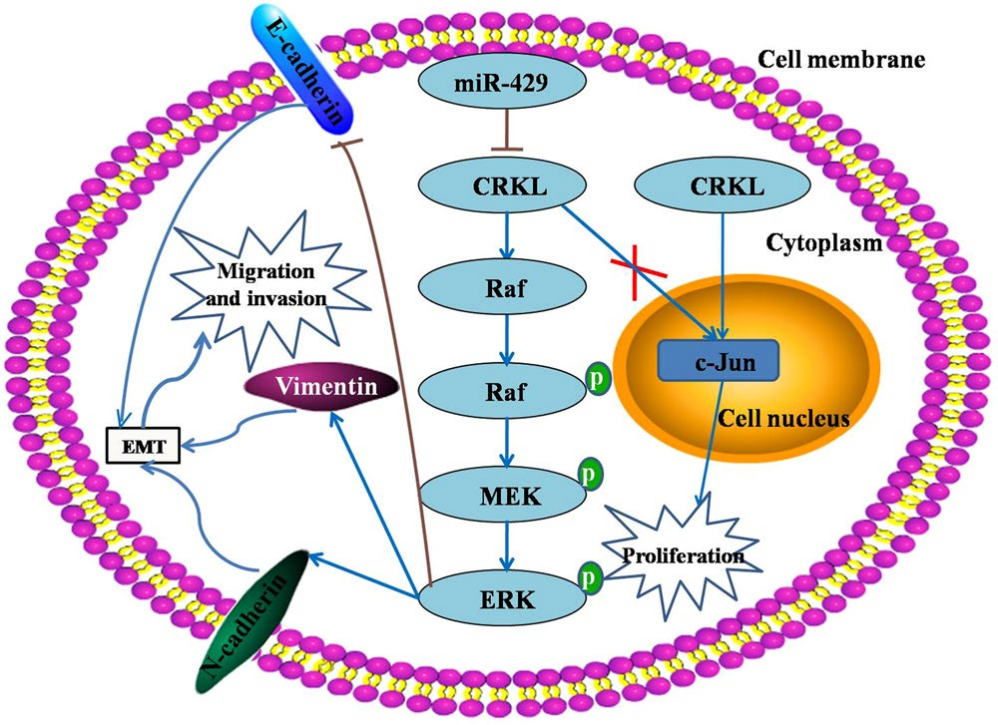
A schematic regulation mechanism of miR-429-CRKL axis on HCC cell malignant behaviours. miR-429 suppresses cell migration and invasion by targeting CRKL *via* Raf/MEK/ERK-EMT pathway; CRKL promotes tumor cell proliferation through upregulating c-Jun; miR-429 could not inhibit tumor cell proliferation through CRKL/c-Jun pathway.

In conclusion, we demonstrated that miR-429 could significantly inhibit HCC cell invasion and metastasis by targeting CRKL, which is a functional target of miR-429. The novel action mechanism was outlined in Fig. 12. miR-429 downregulated CRKL expression at the post-transcriptional protein translation level by directly targeting its 3′-UTR, CRKL downregulation inhibited the expression level of Raf, p-Raf, p-MEK and p-ERK2, and then suppressed migration and invasion through inhibiting EMT by increasing the epithelial marker E-cadherin expression, and decreasing the mesenchymal marker N-cadherin and Vimentin expression; Meanwhile, CRKL promoted tumor cell proliferation through upregulating the expression level of c-Jun; Nevertheless, miR-429 could not inhibit tumor cell proliferation through the CRKL/c-Jun pathway. The newly identified miR-429-CRKL axis provides new insight into the pathogenesis of HCC and represents a potential therapeutic target for diagnosis and treatment of HCC.

## Materials and Methods

### Patients and tissue samples

A total of 12 pairs of frozen human HCC tissues and their coprresponding non-tumor liver tissues were collected from Division of Hepatobiliary and Pancreatic Surgery, Department of Surgery, The Second Affiliated Hospital of Dalian Medical University, Dalian, China. None of the patients had received radiotherapy or chemotherapy before surgery. There were 8 men and 4 women, 5 patients with the age of ≥ 60 years, 7 patients with the age of < 60 years. Tissue specimens were frozen in liquid nitrogen immediately after surgical resection and stored at −80 °C until use. The use of human tissues and study protocol was approved by the Medical Ethics Committee of Dalian Medical University, and informed consent was obtained from all patients. All experiment methods were performed in accordance with the relevant guidelines and regulations.

### Cell culture

Human  hepatocellular carcinoma HepG2 cell was obtained from American Type Culture Collection (ATCC, VA) and cultured in 85% RPMI-1640 (Gibco, USA) medium supplemented with 15% foetal bovine serum (FBS, TransGen, China), 100 U/ml penicillin and 100 U/ml streptomycin (Gibco, USA) in a humidified incubator at 37 °C with 5% CO_2_.

### Plasmid construction

A 2187 bp fragment of the wild-type *CRKL*-3′-UTR containing the two putative potential miR-429-binding sites was amplificated by RT-PCR, inserted into the downstream of a luciferase reporter gene infirefly luciferase expressing vector of psiCHECK-2 with *Xho* I and *Not* I cleavage sites and named as psiCHECK-2-CRKL-3′-UTR-WT. The forward primer and reverse primer were designed as: 5′-CTCGAGGCTCATAGTGAACACAGCAGACCTAGAAATGTAGC-3′ and 5′-GCGCGGCCGCCGGCTGATGCAAGTTTTATTGAGACAATAT-3′, respectively. Site-directed mutagenesis was done to construct psiCHECK-2-CRKL-3′-UTR-MUT1 and -MUT2 by replacing the seed regions of CAGUAUU for mutating the two binding sequences sites with miR-429. Two pairs of primers, forward primer 1 with 5′-CTCGAGGCTCATAGTGAACACAGC-3′, reverse primer 1 with 5′-TTCATGAATACAGCAGTTATCTGCCTGATGGTAATCACATTTAAAG-3′, and forward primer 2 with 5′-CTTTAAATGTGATTACCATCAGGCAGATAACTGCTGTATTCATGAATCT-3′, reverse primer 2 with 5′-TAGCGGCCGCTAAAGGGCAAACAC-3′ were designed for psiCHECK-2-CRKL-3′-UTR-MUT1. Primer 3 with forward and reverse sequences of 5′-CTCGAGGGGATGATGTGGTTTTT-3′ and 5′-ACACTCGATGTATTCTAAGAATATAAGTC ATTATATTATCACTTAATTTTATAGCAC-3′ and primer 4 with forward and reverse sequences of 5′-GTGCTATAAAATTAAGTGATAATAATGACTTATATTCTTAGAATACATCGAGTG-3′ and 5′-GCGCGGCCGCCGGCTGATGCAAG-3′ were for psiCHECK-2-CRKL-3′-UTR-MUT2.

For CRKL knockdown, targeting shRNAs were designed according to *CRKL* sequence (Genbank: NM_005207.3) using Invitrogen, siDirect and Whitehead software. Out of three *CRKL*-specific shRNA double-stranded oligodeoxyribonucleotides, the optimal shRNA for CRKL knockdown was with the sense and antisense sequences of 5′-GTCACAAGGATGAATATAA-3′ and 5′-TTATATTCA TCCTTGTGAC-3′. One non-targeting shRNA with the sense and antisense sequences of 5′-GTTCTCCGAACGTGTCACGT-3′ and 5′-ACGTGACACGTTCGGAGAAC-3′ was used for negative control (NC). The constructed corresponding vectors were named as pGPU6/GFP/Neo-shRNA-CRKL and pGPU6/GFP/Neo-shRNA-NC.

To overexpress CRKL in HepG2 cells, the full-length coding sequence of *CRKL* was first amplificated by RT-PCR using the forward primer 5′-TATCTAGAGCCACCATGTCCTCCGCCAGGTT-3′ and reverse primer 5′-CGTTCGAAGGGCCTCGTTTTCATCTGGGTTT-3′, then cloned into the *BstB* I and *Xba* I sites of PCDH-EF1-MCS-T2A-Puro vector. The recombinant PCDH-EF1-MCS-T2A-Puro-CRKL expression vector was used for overexpressing CRKL in HepG2 cells.

### Oligonucleotide and plasmid transfections

The oligonucleotides of miR-429, miR-NC, LNA-miR-429, LNA-miR-NC and the vectors of PCDH-EF1-MCS-T2A-Puro-CRKL, PCDH-EF1-MCS-T2A-Puro, pGPU6/GFP/Neo-shRNA-CRKL, pGPU6/GFP/Neo-shRNA-NC were transfected into HepG2 cells using Lipofectamine^TM^2000 (Invitrogen, USA) according to manufacturer’s instruction. The cells stably transfected with pGPU6/GFP/Neo-shRNA-CRKL and pGPU6/GFP/Neo-shRNA-NC were screened against 400 μg/ml G418 (Invitrogen, USA) for 21 d. Monoclonal HepG2-shCRKL and HepG2-shNC cells were obtained by limited dilution screening against G418 selection. The cells transiently transfected with miR-429, miR-NC, LNA-miR-429, LNA-miR-NC, PCDH-EF1-MCS-T2A-Puro-CRKL and PCDH-EF1-MCS-T2A-Puro were named as HepG2-miR-429, HepG2-miR-NC, HepG2-LNA-miR-429, HepG2-LNA-miR-NC, HepG2-PCDH-CRKL and HepG2-PCDH, respectively.

### Luciferase reporter gene assay

An expression vector, psiCHECK-2, was used for the luciferase reporter gene assay. The psiCHECK-2 vector provided the constitutive expression of Firefly luciferase as an internal control. 1 × 10^5^ HepG2 cells/well were seeded into a 24-well plate in 1 ml of 85% RPMI-1640 supplemented with 15% FBS without antibiotic and cultured in a humidified environment at 37 °C with 5% CO_2_ for 24 h. HepG2 cells were co-transfected with 50 mM miR-429 mimic or negative control and 500 ng of psiCHECK-2-CRKL-3′-UTR-WT, psiCHECK-2-CRKL-3′-UTR-WT1, psiCHECK-2-CRKL-3′-UTR-WT2, psiCHECK-2-CRKL-3′-UTR-MUT1 or psiCHECK-2-CRKL-3′-UTR-MUT2, respectively. The cells were collected in 48 h after transfection and detected by using a Dual-Luciferase Reporter Assay System (Promega, CA, USA). The cells in each well were then washed with PBS, lysed in 100 μl passive lysis buffer (PLB) with gentle shaking for 15 min at room temperature (RT). The lysate was transferred into a luminometer tube, mixed well with 50 μl of luciferase assay reagent II (LAR II) first for Firefly luciferase activity assay and then mixed with 50 μl of stop reagent for Renilla luciferase activity assay. Luciferase activity was determined using a GloMax fluorescence reader (Promega, CA, USA).

### MTT and colony forming assays

The influences of miR-429 and CRKL level changes on HepG2 proliferation was determined by MTT assay. The cells from each group were seeded into a 96-well plate at 3 × 10^3^ cells/well in 100 μl RPMI-1640 supplemented with 15% FBS, incubated at 37 °C, 5% CO_2_ for 24, 48, 72 and 96 h separately, incubated in MTT solution (5 mg/ml) by replacing culture medium at 37 °C, 5% CO_2_ for 4 h in darkness. The supernatants were then removed and 150 μl DMSO was added to dissolve for formazan crystals. The absorbance at 492 nm were measured using a microplate reader (Thermo, USA) for cell density quantification. Results were obtained from triplicate measurements.

The deregulation effects of miR-429 and CRKL on HepG2 colony forming ability was performed by colony formation assay. The dissociated 1000 cells in 2 ml RPMI-1640 supplemented with 15% FBS were seeded in 6-well plate and then kept at 37 °C with 5% CO_2_ for 10 d until visible cell colonies appeared. The colonies were washed with PBS, fixed with methanol for 30 min and stained with 0.05% crystal violet for 1 h at RT. The colonies were observed and larger than 50 cells were counted using an upright light microscope (Olympus, Japan) with magnification of 100x. Triplicate experiments were performed for each assay.

### *In vitro* cell migration and invasion assays

The effect of miR-429 and CRKL deregulations on the migration and invasion abilities of HepG2 cells were examined using 24-well-plate transwell chamber assay. Briefly, 1 × 10^4^ cells in 200 μl serum-free RPMI-1640 were seeded onto the upper compartment of transwell with 8 μm pore size polycarbonate filters (Corning, USA). The chambers were then placed into 24-well plates containing 600 μl RPMI-1640 with 20% FBS and incubated for 48 h at 37 °C with 5% CO_2_. For invasion assay, the inserts were coated with 50 μl extracellular matrix gel (ECM, Sigma, USA) which 1:8 dilution with RPMI-1640, and incubated at 37 °C for 1 h. 1 × 10^4^ cells in 200 μl serum-free RPMI-1640 were seeded onto the upper compartment of transwell. The chambers were then placed into 24-well plates containing 600 μl RPMI-1640 with 20% FBS and incubated for 48 h at 37 °C with 5% CO_2_. The non-migrated and non-invaded cells on the upper surface of the insert were removed by swabbing, the migrated and invaded cells onto the lower surface were fixed with methanol for 30 min, stained with 0.5% crystal violet for 1 h, washed with PBS, counted using an upright light microscope (Olympus, Japan) with 100x. Five field views were randomly counted and averaged.

### Extracellular matrix adhesion assay

The effect of miR-429 and CRKL deregulations on the adhesion capacity of HepG2 to fibronectin (FN, Millipore, USA) was examined. Each well of a 96-well plate were first coated with 50 μl 50 μg/ml FN by incubating at RT for 1 h and blocked with 50 μl 1% BSA for 1 h at 37 °C. 1 × 10^3^ cells in 100 μl RPMI-1640 containing 15% FBS were seeded into each well of a 96-well plate and cultured at 37 °C, 5% CO_2_ for 2 h. The non-adherent cells were washed with PBS, incubated in 5 mg/ml MTT solution at 37 °C with 5% CO_2_ in darkness for 4 h. Then, 150 μl DMSO was added into each well to dissolve formazan. The absorbance at 492 nm was measured using a microplate reader (Thermo, USA) for cell number quantification. The results were averaged from triplicate independent experiments.

### *In situ* cell adhesion potential to lymph node (LN) assay

The effect of miR-429 and CRKL on HepG2 adhesion ability to LNwas examined by *in situ* cell adhesion assay. Fresh LNs taken from 615 mice were frozen at −20 °C, embedded by optimum cutting temperature compound (OCT) and sectioned into 10 μm slices. The frozen LN slices were merged in 200 μl RPMI 1640 supplemented with 15% FBS containing 2 × 10^5^ cells from each group, incubated at 37 °C with 5% CO_2_ for 24 h, washed with ice-cold PBS for 3 times, fixed in 95% ethanol for 5 min, washed with distilled water for 3 s and stained with hematoxylin eosin (HE). The number of adherent cells was counted using an upright light microscope (Olympus, Japan) with 100 × by randomly selecting five fields.

### F-actin cytoskeleton staining assay

FITC-Phalloidinstaining assay was performed to investigate the influence of miR-429 and CRKL on the cytoskeleton structure of HepG2 cells. The 0.17 mm thick round cover glasses with 25 mm in diameter were placed into 6-well plates, then 3 × 10^4^ cells from each group were seeded into cover glasses and cultured for 24 h at 37 °C in humidified incubator with 5% CO_2_. The cover glasses were then washed with PBS for 2 times, fixed in 4% paraformaldehyde for 10 min at RT, washed with PBS for 3 times, permeabilized with acetone, and continuously incubated in 200 μl 400 nM Phalloidin-FITC containing 1% BSA for 30 min in the dark at RT. The cover glasses were washed with PBS for 3 times and immediately imaged for examining the actin cytoskeleton organization and filaments using an oli microscope (Olympus, Japan) at five randomly selected visual fields of 1000x.

### Quantitative real-time RT-PCR(qRT-PCR) assay

Total RNA was extracted from each group cells using Trizol^TM^ reagent (Invitrogen, USA) and reversely transcribed into cDNA using PrimeScript^TM^ RT Kit with gDNA Eraser (Takara, Japan). qRT-PCR was then performed using FastStart universal SYBR Green Master (ROX) (Roche, USA) with an Applied Biosystems StepOne^TM^ Real-Time PCR System (Life, USA). snRNA U6 and β-actin (*ACTB*) were used as internal references for miR-429 and mRNA, respectively. The relative expression levels of miR-429 and targeting genes among different cell lines, and in paired tumor and non-tumor tissues were comapred using 2^−△△CT^ method. The specific primers for *CRKL, c-Jun, E-cadherin, N-cadherin, Vimentin* and *ACTB* were provided in Table 1.

**Table 1:** Synthesized sequences of primers for targeting genes.

Targeting gene	Primer sequence
*CRKL*	F:5′-GTGCTTATGACAAGACTGCCT-3′
	R:5′-CACTCGTTTTCATCTGGGTTT-3′
*c-Jun*	F:5′-GCTGCCTCCAAGTGCCGAAA -3′
	R:5′-TAAGCTGTGCCACCTGTTCCC -3′
*E-cadherin*	F:5′-TGAGGGGTTAAGCACAACAGCAA-3′
	R:5′-GCCATCGTTGTTCACTGGAT-3′
*N-cadherin*	F:5′-ATGCCCCTCAAGTGTTACCTC-3′
	R:5′-CAAAATCACCATTAAGCCGAGT-3′
*Vimentin*	F:5′-CTTGACATTGAGATTGCCACC-3′
	R:5′-CCATCTCTAGTTTCAACCGTCT-3′
*ACTB*	F:5′-AGGCCAACCGCGAGAAG-3′
	R:5′-ACAGCCTGGATAGCAACGTACA-3′

### Western blotting (WB) assay

Total protein was extracted from each group cells using RIPA buffer (50 mM pH 8.0Tris-HCl, 150 mM NaCl, 1% Triton X-100, 0.5% sodium deoxycholate, 0.1% SDS in the presences of 1 mM Na_3_VO_4_, 1 μg/ml leupeptin and 0.5 mM PMSF). The supernatant was collected by centrifugation at 12000 rpm for 15 min at 4 °C. Equal amounts of each protein sample determined by Bradford assay were boiled for 5 min in loading buffer and separated by 10% SDS-PAGE. The protein bands were transferred onto nitrocellulose (NC) membrane (Millipore, Merck), blocked with 5% (w/v) skim milk (BD, USA) in TBST (pH 7.5; 100 mM NaCl, 50 mM Tris and 0.1% Tween-20) for 3 h at RT and then incubated with primary antibodies at 4 °C overnight. The primary antibodies were CRKL (1:2000, Genex, USA), Ras (1:500, Ruiying, China), Raf (1:500, Cell Signaling, USA), p-Raf (1:500, pTyr341, Cell Signaling, USA), p-MEK1/2 (1:500, pSer217/221, Cell Signaling, USA), ERK1/2 (1:1000, Cell Signaling, USA), p-ERK1/2 (1:500, pThr202/pTyr204, Cell Signaling, USA), c-Jun (1:500, Abbkine, USA), p-c-Jun (1:500, pSer73, Abbkine, USA), p-c-Jun (1:500, pSer243, Abbkine, USA), JNK (1:500, Abbkine, USA), p-JNK (1:500, pThr183/185, Abbkine, USA), ACTB (1:4000, Sanying, China). The NC membrane was then washed with TBST for 3 × 10 min, incubated with the secondary antibody conjugated for 3 h at RT and washed again with TBST for 3 × 10 min. Protein bands were visualized by ECL (Advansta, USA) and analyzed by Bio-Rad ChemiDoc^TM^ MP system (Bio-Rad, USA).

### Data processing and statistical analysis

The data were processed as mean ± standard deviations of at least three independent experiments. The differences between groups were evaluated by unpaired Student’s *t*-test analysis. Differences with the values of *P* ≤ 0.05 were significant.
